# Non-coding RNAs fine-tune the balance between plant growth and abiotic stress tolerance

**DOI:** 10.3389/fpls.2022.965745

**Published:** 2022-10-12

**Authors:** Yingying Zhang, Ye Zhou, Weimin Zhu, Junzhong Liu, Fang Cheng

**Affiliations:** ^1^ Shanghai Key Laboratory of Protected Horticulture Technology, The Protected Horticulture Institute, Shanghai Academy of Agricultural Sciences, Shanghai, China; ^2^ State Key Laboratory of Conservation and Utilization of Bio-Resources in Yunnan and Center for Life Sciences, School of Life Sciences, Yunnan University, Kunming, China

**Keywords:** non-coding RNAs, trade-off, abiotic stress responses, phytohormone, stress memory

## Abstract

To survive in adverse environmental conditions, plants have evolved sophisticated genetic and epigenetic regulatory mechanisms to balance their growth and abiotic stress tolerance. An increasing number of non-coding RNAs (ncRNAs), including small RNAs (sRNAs) and long non-coding RNAs (lncRNAs) have been identified as essential regulators which enable plants to coordinate multiple aspects of growth and responses to environmental stresses through modulating the expression of target genes at both the transcriptional and posttranscriptional levels. In this review, we summarize recent advances in understanding ncRNAs-mediated prioritization towards plant growth or tolerance to abiotic stresses, especially to cold, heat, drought and salt stresses. We highlight the diverse roles of evolutionally conserved microRNAs (miRNAs) and small interfering RNAs (siRNAs), and the underlying phytohormone-based signaling crosstalk in regulating the balance between plant growth and abiotic stress tolerance. We also review current discoveries regarding the potential roles of ncRNAs in stress memory in plants, which offer their descendants the potential for better fitness. Future ncRNAs-based breeding strategies are proposed to optimize the balance between growth and stress tolerance to maximize crop yield under the changing climate.

## Introduction

Owing to the sessile nature, plants are exposed to diverse abiotic stresses due to climate change, such as drought, salinity, and extreme temperatures (cold and heat), high light, ozone, which pose a serious threat to crop productivity worldwide (Lobell et al., 2011; Hirabayashi et al., 2013; Hasan et al., 2021; Barczak-Brzyzek et al., 2022). To cope with stress conditions, plants have evolved various stress response mechanisms, including stress avoidance or escape, tolerance, and recovery mechanisms (Fang and Xiong, 2015). Upon the perception of stress signals, plant cells activate an assortment of downstream responses to restore cellular RNA, proteins, metabolism and reactive oxygen species (ROS) homeostasis, such as the initiation of calcium signaling, mitogen-activated protein kinase (MAPK) activation, the triggering of phosphoprotein cascades, the generation of various ROS, accumulation of osmotic regulating substances, changes in phospholipid composition, and global transcriptional reprogramming (Gong et al., 2020; Zhang et al., 2022). However, due to nutrient restriction and reallocation, activating these abiotic stress responses frequently occur at the cost of a reduction in plant growth, productivity and quality. Despite our increasing knowledge on plant abiotic stress responses, how plants maintain a balance between their growth and stress responses is still poorly understood. Growth-stress response trade-offs are thought to determine the prioritization towards either growth or abiotic stress responses, which depend on the interaction between stress and growth regulatory pathways. In order to maximize crop yield to meet increasing food demand, better understanding of these trade-offs is required for developing breeding strategies to re-design or optimize the balance between growth and abiotic stress resistance, thus optimizing crop breeding for specific or general abiotic stress conditions.

Non-coding RNAs (ncRNAs) are a class of RNAs which are transcribed in the genome but do not encode proteins. Recent genome-wide transcriptome analyses have revealed that in addition to the well-known rRNA, tRNA, small nuclear RNAs (snRNAs) and small nucleolar RNAs (snoRNAs), a large number of other ncRNAs, including 18-30 nucleotide (nt) small RNAs (sRNAs), medium-sized ncRNAs (31-200 nt), and long non-coding RNAs (lncRNAs) (> 200 nt), are expressed in plants. These ncRNAs differ in their biosynthesis and modes of action on target genes. Despite the differences, the regulatory pathways of ncRNAs are interconnected and either synergistically or antagonistically regulate the expression of target genes or protect the genome from both external and internal threats (Ghildiyal and Zamore, 2009). ncRNAs regulate diverse cellular processes in chromatin remodeling, epigenetic memory, transcription, turnover, RNA splicing, editing and translation, and play vital roles in maintaining genomic stability, plant growth and development, senescence, and plant responses to biological and abiotic stresses (Borges and Martienssen, 2015; Chekanova, 2015; Song et al., 2019; Yu et al., 2019; Jampala et al., 2021). At present, there are many excellent reviews regarding the roles of ncRNAs in plant development, immunity and abiotic stress (Jatan and Lata, 2019; Song et al., 2019; Yu et al., 2019; Song et al., 2021a; Tang et al., 2021). However, the roles and mechanisms of ncRNAs in the trade-off between growth and abiotic stress responses remain largely scattered and fragmented.

In this review, we summarize recent findings and the current progress on ncRNAs-mediated prioritization towards plant growth or tolerance to abiotic stresses, with a focus on hormone-based signaling crosstalk. We further highlight the potential roles of ncRNAs in the interplay between growth and stress responses in plants during transgenerational memory. We then propose perspectives in ncRNAs-based breeding strategies to make growth and stress tolerance compatible to maximize crop yield.

## Overview of plant small RNAs

sRNAs in plants can be divided into microRNAs (miRNAs) and small interfering RNAs (siRNAs) according to their synthesis pathways and modes of function. miRNAs are non-coding small RNAs with a length of 20-24 nt and are encoded by *MIR* genes which are usually located in intergenic regions with some found in the intronic sequences of protein coding genes (Song et al., 2019; Yu et al., 2019). The plant *MIR* genes may be generated by spontaneous evolution, genic inverted repeats, mutation, *MIR* family expansion and neofunctionalization, and functional divergence (Baldrich et al., 2018) and mostly originate from chlorophyte algae (e.g., *Chlamydomonas reinhardtii*) (Deng et al., 2022). miRNAs are usually formed by Dicer-like protein 1 (DCL1)-mediated cleavage of precursors, possess a stem-loop structure, and mainly regulate the expression of target genes at the post-transcriptional level (Voinnet, 2009; Rogers and Chen, 2013). siRNAs are processed from long double-stranded RNAs (dsRNAs), which depends on the activity of DCL2-4. siRNAs mediate transcriptional gene silencing (TGS) through cytosine methylation at homologous DNA loci or posttranscriptional gene silencing (PTGS) by targeting mRNA for slicing or translational inhibition. According to their origin and biogenesis pathway, plant endogenous siRNAs can be further divided into heterochromatic siRNAs (hc-siRNAs), phased secondary siRNAs (phasiRNAs), natural antisense transcripts siRNAs (nat-siRNAs), and so forth (Borges and Martienssen, 2015).

Hc-siRNAs are a class of 24 nt sRNAs, which mainly play vital roles in RNA-directed DNA methylation (RdDM), maintenance of genome stability and regulation of gene expression (Law and Jacobsen, 2010; Zhang et al., 2018a). PhasiRNAs are generated from phasiRNA-generating loci (*PHAS*) in a phased manner upon miRNA-mediated cleavage (Fei et al., 2013; Sakurai et al., 2021; Yoshikawa et al., 2021) and drive transcriptional or post-transcriptional gene silencing (Fei et al., 2013). Some phasiRNAs such as *trans*-acting small interfering RNAs (tasiRNAs), can function in *trans* to modulate the expression of target mRNAs or non-coding transcripts (Allen et al., 2005; Montgomery et al., 2008; Fei et al., 2013). The precursors of nat-siRNAs are derived from overlapping sense and antisense transcripts that share the same spatio-temporal expression pattern. Natural antisense transcript pairs form dsRNAs, which serve as the substrate for the RNA silencing machinery and are processed into nat-siRNAs (Zhang et al., 2013).

## miRNAs modulate the trade-off between plant growth and abiotic stress responses

Plant miRNAs regulate the expression of target genes at the transcriptional and post-transcriptional levels by mRNA cleavage, translation inhibition, or RdDM. Various miRNAs have been shown to regulate plant development, including cell division, cell proliferation, and vegetative and reproductive growth (Mallory et al., 2004; Guo et al., 2005; Reyes and Chua, 2007; Rogers and Chen, 2013; Song et al., 2019). For example, the miR156/157/529 superfamily is a highly abundant and conserved miRNA family in the plant kingdom. miR156/157/529 participates in endosperm nuclear division (Zhao et al., 2018), both the vegetative phase and floral transition (Wu et al., 2009; Guo et al., 2017), plant architecture including plant height, tiller number, panicle architecture, and grain size (Jiao et al., 2010; Yue et al., 2017; Yan et al., 2021), and seed dormancy (Miao et al., 2020). Specifically, miR165/166 regulates the root xylem formation and vascular acclimation (Ramachandran et al., 2018); miR167 influences embryonic and seed development (Yao et al., 2019); miR319 is involved in secondary cell wall biosynthesis (Sun et al., 2017), and miR393 participates in soybean (*Glycine max* L.) nodulation (Cai et al., 2017). As sessile organisms, plant growth and development are often affected by various environmental factors, and miRNAs are implicated in plant responses to adverse conditions (Singh et al., 2021). In recent years, high-throughput small RNA sequencing technology has expedited our identification of miRNAs that are involved in plant abiotic stress (Zhong et al., 2013; Liu et al., 2015; Xie et al., 2015; Fu et al., 2017). miRNAs and their target genes form a complex regulatory network with other stress response-related genes (Song et al., 2019; Yu et al., 2019).

Due to resource restrictions, the trade-off between growth and defense against adversity is generally considered as the best strategy for plant to achieve optimal fitness (Huot et al., 2014; He et al., 2022). To ensure survival and successful reproduction, plants have developed complicated regulatory machineries. Plants can accelerate growth to complete flowering and fruiting, thus avoiding extreme environments (Fang and Xiong, 2015); or grow slowly to accumulate more energy and build defense barriers, thereby defending against unfavorable environments (Gong et al., 2020). The adaption to stress conditions in plants is often achieved at the cost of phenotypic changes such as alterations in flowering time, floral organ development, fertility, seed-set, seed trait, plant architecture, root structure, and leaf morphology.

Here we briefly summarize the function of miRNAs in plant responses to several abiotic stresses including heat, cold, drought, and salt stresses, and then discuss the role of these miRNAs in the trade-off between plant growth and abiotic stress responses.

### miRNAs modulate plant abiotic stress responses

The global warming-induced increase in average global temperature and recurrent extreme heat stress have become one of the major threats to food security (Lobell and Field, 2007). Heat stress has multiple effects on plant growth, development, and reproduction (Lobell et al., 2013; Hancock et al., 2014; Sánchez et al., 2014; Zhang et al., 2019a). The conserved heat-responsive miRNAs in different plant species such as miR156, miR159, miR160, miR166, miR319, miR390, miR393, miR396, miR398, miR408, and miR827 have been summarized (Liu et al., 2015; Zhao et al., 2016; Gahlaut et al., 2018) (**Table 1**). For example, heat stress induces the expression of miR156, which promotes the sustained expression of heat stress responsive gene, thereby maintaining the acquired thermotolerance in *Arabidopsis*. More importantly, miR156-mediated repressions of *SQUAMOSA PROMOTER BINDING-LIKE 2* (*SPL2*) and *SPL11* are critical for the somatic heat stress memory (Stief et al., 2014). Heat stress induces the expression of miR160 in *Arabidopsis*, barley (*Hordeum vulgare*), celery (*Apium graveolens*), and cotton (*Gossypium hirsutum*) (Kruszka et al., 2014; Li et al., 2014; Ding et al., 2017; Lin et al., 2018). Overexpression of miR160 improves seed germination, rachis growth and seedling survival in *Arabidopsis* by repressing the expression of its targets *AUXIN RESPONSE TRANSCRIPTION FACTOR 10/16/17* (*ARF10/16/17*) under heat stress, which may contribute to thermotolerance through the activation of *HEAT SHOCK PROTEIN* (*HSP*) genes (Lin et al., 2018). However, in cotton, miR160 inhibits anther dehiscence by inhibiting *ARF10/17* expression under high temperature stress (Ding et al., 2017). These reports suggest that miR160 may have a different function in different species. miR396 is also responsive to heat stress. The heterologous expression of sunflower (*Helianthus annuus*) miR396-resistant *HaWRKY6* in *Arabidopsis* decreases plant thermotolerance (Giacomelli et al., 2012). Heat stress induced up-regulation of miR398 represses the expression of its target genes *COPPER/ZINC SUPEROXIDE DISMUTASE1* (*CSD1*), *CSD2* and *COPPER CHAPERONE FOR SOD1* (*CCS*) to promote the accumulation of ROS, which contributes to the induction of heat stress transcription factors (HSFs) and HSPs that are required for *Arabidopsis* thermotolerance (Guan et al., 2013). Interestingly, a recent study shows that the processing of pri-miR398b/c and miR398 are repressed by the natural antisense transcripts of *MIR398b*/*c*, which leads to the up-regulation of *CSD1* and high temperature sensitivity (Li et al., 2020d).

**Table 1:** Functions of conversed miRNAs in plant growth, development, and abiotic stresses.

miRNAs	Targets	Functions in growth and development	Functions in abiotic stresses (heat, cold, drought and salt)
miR156^★^	*SPLs;* *IPA1;* *WD40*	Plant architecture (Jiao et al., 2010); Vegetative phase and floral transition (Wu et al., 2009; Guo et al., 2017); Endosperm nuclear division (Zhao et al., 2018); Plant height, tiller number, panicle architecture and grain size (Jiao et al., 2010; Yue et al., 2017; Yan et al., 2021)	Heat stress memory (Stief et al., 2014); Drought tolerance (Arshad et al., 2017; Arshad et al., 2018; Kang et al., 2020); Drought recovery (Visentin et al., 2020); Cold resistance (Zhou and Tang, 2019); Salt tolerance in tobacco (Kang et al., 2020); Salt sensitive in apple (Ma et al., 2021); Tolerance to low temperature, salt and dehydration stress in rice (Yang et al., 2012)
miR159	*MYBs;* *TCPs*	Seed germination (Reyes and Chua, 2007); Anther development (Wang et al., 2012)	Heat sensitive (Wang et al., 2012)
miR160	*ARF10/16/17*	Anther development (Ding et al., 2017); Seed germination, hypocotyl growth, and rachis growth (Lin et al., 2018); Adventitious root development (Shen et al., 2022)	Heat sensitivity in cotton (Ding et al., 2017); Thermotolerance in *Arabidopsis* (Lin et al., 2018); Drought tolerance (Shen et al., 2022)
miR165/166^★^	*HD-ZIP III*	Root xylem formation and vascular acclimation (Ramachandran et al., 2018); Leaf and stem xylem development (Zhang et al., 2018b)	Drought and cold sensitivity (Yan et al., 2016); Drought, heat and heat sensitivity (Li et al., 2020a)
miR167	*ARF, IAR3*	Embryonic and seed development; (Yao et al., 2019); Reproduction, root architecture (Kinoshita et al., 2012)	Drought sensitive (Kinoshita et al., 2012)
miR169	*NF-YA*		Drought sensitive (Ni et al., 2013)
miR172^★^	*AP2; IDS1; TOE1/2*	Floral transition (Li et al., 2016)	Salt tolerance (Li et al., 2016; Cheng et al., 2021); Drought tolerance (Li et al., 2016)
miR319^★^	*TCPs; GAMYB*	Secondary cell wall biosynthesis (Sun et al., 2017)	Cold tolerance (Yang et al., 2013); Salt tolerance (Zhou et al., 2013; Liu et al., 2019b); Drought tolerance (Zhou et al., 2013); Chilling and heat tolerance (Shi et al., 2019)
miR390	*TAS3; ARF2/3/4*	Lateral root growth (Marin et al., 2010; He et al., 2018; Lu et al., 2018)	Salt stress (He et al., 2018)
miR393^★^	*AFB/TIR1*	Root system architecture (Lu et al., 2018); Tiller and flowering (Xia et al., 2012); Soybean nodulation (Cai et al., 2017)	Drought, salt and heat stress tolerance in creeping bentgrass (Zhao et al., 2019); Cold tolerance in switchgrass (Liu et al., 2017); Salt and drought sensitive in rice (Xia et al., 2012)
miR396	*GRF; bHLH;* *HaWRKY6; ACO*	Grain length, grain width, and grain weight (Chen et al., 2019)	Salt stress sensitive in *Arabidopsis* and rice (Gao et al., 2010); Heat stress tolerance (Giacomelli et al., 2012); Salt tolerance in creeping bentgrass (Yuan et al., 2019); Cold tolerance (Zhang et al., 2016; Chen et al., 2019)
miR398	*CSD1/2;* *CCS1;* *AGL51/52/78*	ROS homeostasis (He et al., 2021)	Heat stress tolerance (Guan et al., 2013); Thermotolerance (Li et al., 2020b); Salt sensitivity (He et al., 2021)
miR399^★^	*PHO2*	Phosphate signaling pathway, maintenance of phosphate homeostasis (Peng et al., 2021)	Freezing tolerance (Peng et al., 2021); Drought sensitive (Baek et al., 2016); Salt tolerance (Baek et al., 2016)
miR408^★^	*LAC3/LAC12/LAC13*	Mediates a coordinated response to light and copper (Ma et al., 2015a)	Salt and cold tolerance, drought sensitive (Ma et al., 2015a)
miR528	*AO; PPO; AAO; LAC; POD; SOD*	ROS homeostasis (Yuan et al., 2015)	Salt tolerance (Yuan et al., 2015)
miR535^★^	*SPLs*	Seedling growth (Sun et al., 2020)	Salt stress sensitive (Yue et al., 2020); Dehydration sensitive (Yue et al., 2020); Cold stress sensitive (Sun et al., 2020)

^★^The miRNAs involved in multiple abiotic stresses responses.

Cold stress is one of the important limiting factors of plant growth, development and geographical distribution. It mainly affects plant cell enzyme activity, membrane systems and cell water, leading to disorders of cell metabolism and even programmed cell death (Yadav, 2010; Ding et al., 2019). miRNAs may regulate cold stress tolerance through regulating stress-related signal transduction pathways or modulating the expression of cold responsive transcription factors such as *C-REPEAT BINDING FACTORS* (*CBFs*) and *INDUCER OF CBF EXPRESSION1* (*ICE1*), the core regulators in cold acclimation (Chinnusamy et al., 2007). Some conserved miRNAs, such as miR156, miR165/166, miR319, miR393, miR396, miR399, miR408, and miR535 have been reported to be involved in plant responses to cold stress (**Table 1**). The miR156-SPL module has been reported to enhance the cell viability and growth rate of rice, *Arabidopsis* and pine cells under low temperature stress, and improve cold tolerance (Zhou and Tang, 2019). The expression of miR156 and *SPL9* are both induced by freezing treatment, and transgenic *Arabidopsis* overexpressing *SPL9* shows freezing tolerance through SPL-directed activation of *CBF2* (Zhao et al., 2022). Os-miR156 represses OsSPL3-mediated activation of OsWRKY71, thereby releasing OsMYB2 from repression by OsWRKY71. OsMYB2 positively regulates rice tolerance to low temperature, salt, and dehydration stress by activating the expression of stress-responsive genes (Yang et al., 2012). In tomato, overexpression of sha-miR319d (*Solanum habrochaites* microRNA319d) also increases plant chilling and heat tolerance, which may be caused by inhibiting the expression of the target gene *GAMYB-like 1* (Shi et al., 2019). Overexpression of tae-miR399 from the winter wheat cultivar (*Triticum aestivum*) in *Arabidopsis* represses the expression of *AtUBC24*, thereby inhibiting the AtUBC24-mediated degradation of AtICE1 protein. The increased expression of AtICE1 induces the CBFs signaling pathway, resulting in the activation of antioxidant enzymes to enhance plant chilling and freezing tolerance (Peng et al., 2021). Overexpression of miR408, which is highly conserved in terrestrial plants, leads to increased resistance against cold, salt, and oxidative stresses, but causes an enhanced sensitivity to drought and osmotic stress in *Arabidopsis* (Ma et al., 2015a).

Drought is another detrimental environmental factor for plant growth and development. A large number of miRNAs have been shown to participate in drought stress responses (Ding et al., 2013; Zhang, 2015; Shriram et al., 2016; Li et al., 2017). miR167 is repressed by high osmotic stress. Subsequently the mRNA level of its target *IAA-ALA RESISTANT3* (*IAR3*) increases, which positively regulates plant drought tolerance by changing root architecture in *Arabidopsis* (Kinoshita et al., 2012). *GmNFYA3* in soybean, a target gene of miR169, is also a positive regulator in plant drought tolerance (Ni et al., 2013). The Os-miR166-HOMEODOMAIN CONTAINING PROTEIN4 (OsHB4) module was also shown to negatively regulate plant drought resistance by altering leaf and stem xylem development (Zhang et al., 2018b). These results suggest that miR166, miR167, and miR169 may negatively regulate plant drought stress response. miR156 is induced by drought stress and was found to promote drought tolerance in alfalfa (*Medicago sativa*) through silencing *SPL13* and *WD40-2*. Observed effects include reduced water loss, enhanced root growth, stomatal conductance, chlorophyll content and photosynthetic carbon assimilation (Arshad et al., 2017; Arshad et al., 2018). In apple (*Malus domestica*), miR160 cleaves *MdARF17* and relieves MdARF17-mediated inhibition of *MdHYL1* expression. MdARF17-MdHYL1 form a positive feedback loop to regulate the stability of Mdm-miR160, which improves plant drought resistance by promoting adventitious root development and increasing the root-shoot ratio (Shen et al., 2022). As well as the miRNAs mentioned above, many other conserved or non-conserved miRNAs modulate plant drought resistance (Ding et al., 2013; Zhang, 2015; Shriram et al., 2016; Li et al., 2017).

About 20% of the world’s irrigated land are affected by salinity (Morton et al., 2019). Salt stress influences the osmotic status of cells, causing serious ion injury and nutrient imbalance, which severely limits plants growth and development (Gong et al., 2020). Different miRNA families have been shown to modulate plant salt tolerance (Zhang, 2015; Song et al., 2019; Yu et al., 2019). The miR156-SPL module regulates salt stress tolerance in apple by directly activating the expression of *MdWRKY100* (Ma et al., 2021). Interestingly, subtle manipulation of maize (*Zea mays*) miR156c expression in tobacco improves salt and drought tolerance without changes in plant architecture (Kang et al., 2020), suggesting that miR156 can be genetically engineered to improve plant salt stress tolerance. miR172s are positive regulators of salt tolerance in *Arabidopsis*, rice, and wheat. The miR172a/b- INDETERMINATE SPIKELET1 (IDS1) module contributes to plant salt tolerance by maintaining ROS homeostasis during salt stress (Cheng et al., 2021). Os-miR396c is decreased during salt stress and constitutive overexpression of Os-miR396c in both *Arabidopsis* and rice inhibit their tolerance to salt (Gao et al., 2010). However, transgenic creeping bentgrass overexpressing Os-miR396c showed enhanced salt tolerance (Yuan et al., 2019). The opposite responses to salt stress in different plant species suggest a species-specific function of miR396.In addition to cold tolerance, miR399 also participates in the plant response to salt and drought stress. For example, *Arabidopsis* seedlings overexpressing miR399f show hypersensitivity to drought stress but enhanced tolerance to salinity (Baek et al., 2016). Os-miR319a, Os-miR393a, and Os-miR535 have also been shown to regulate resistance against both drought and salt stresses (Zhou et al., 2013; Zhao et al., 2019; Yue et al., 2020). Since both drought stress and salt stress can cause osmotic stress, miRNAs may contribute to drought and salt stress tolerance through modulating plant responses to osmotic stress. The miRNAs involved in multiple abiotic stresses responses are summarized in **Table 1**.

### miRNAs modulate the growth-stress responses balance

Many miRNAs have been reported to regulate plant growth in some plant species and modulate plant abiotic stress responses in other species, such as miR156, miR159, miR160, miR165/166, miR167, miR169, miR172, miR319, miR390, miR393, miR396, miR398, miR399, miR408, miR528, and miR535 (**Table 1**). Research regarding the role of miRNAs in the trade-off between growth and abiotic stress tolerance in the same species is relatively rare.

Accumulating evidence reveals that the miR156-SPLs module plays important roles in balancing plant growth and fitness for abiotic stress. Transgenic tobacco plants expressing Zm-miR156c show improved salt and drought tolerance, featured by vigorous growth, increased biomass, and enhanced antioxidant capacity; however, under normal growth conditions, the constitutive overexpression of Zm-miR156c results in slow growth, increased branching, and later flowering (Kang et al., 2020). Interestingly, transgenic tobacco plants expressing Zm-miR156c driven by the stress-inducible promoter Zm-Rab17 display enhanced drought and salt tolerance without obvious developmental changes (Kang et al., 2020). Transgenic rice plants overexpressing wheat Ta-miR159 display delayed heading time and male sterility, but are less tolerant to heat stress relative to the wild type (Wang et al., 2012). *Arabidopsis* seedlings overexpressing miR160 show improved seed germination and survival rates under heat stress (Lin et al., 2018). In apple, the miR160-MdARF17-MdHYL1 module enhances drought tolerance by promoting adventitious root development (Shen et al., 2022). miR166 also participates in the growth-defense trade-off in rice, maize and tomato. The knockdown of rice miR166 results in drought resistance at the cost of alterations in leaf and stem xylem development (Zhang et al., 2018b). In maize, inactivation of miR166 enhances plant tolerance to heat, drought and salt stress, along with various morphological changes, including inferior yield-related traits, rolled leaves, a smaller tassel size, decreased vascular bundles and metaxylem vessels, all of which may be associated with a decreased indole acetic acid (IAA) content and a high abscisic acid (ABA) content (Li et al., 2020a). In tomato, miR166 has been shown to be a cold-inducible switch that controlled parthenogenesis through adjusting the accumulation of *SlHB15A*, a HD-ZipIII transcription factor which regulated fruiting by controlling the synthesis of IAA, gibberellic acid (GA), and ethylene (ET) (Clepet et al., 2021).The miR167-IAR3 module confers drought tolerance and changes in root architecture in *Arabidopsis* (Kinoshita et al., 2012). Overexpression of soybean gma-miR172c in *Arabidopsis* accelerates flowering and confers tolerance to salt and drought stresses (Li et al., 2016). Transgenic creeping bentgrass overexpressing Os-miR319a show enhanced salt and drought tolerance as well as altered leaf development (Zhou et al., 2013). Transgenic rice plants overexpressing miR319, or by silencing its target gene *OsPCF5/8*, display enhanced cold tolerance after chilling acclimation, which may be associated with the increase in leaf growth and veins (Yang et al., 2013). Together, these studies demonstrate that miR156, miR159, miR160, miR166, miR167, miR172, and miR319 play important roles in the trade-off between growth and abiotic stress tolerance.

**Figure 1:**
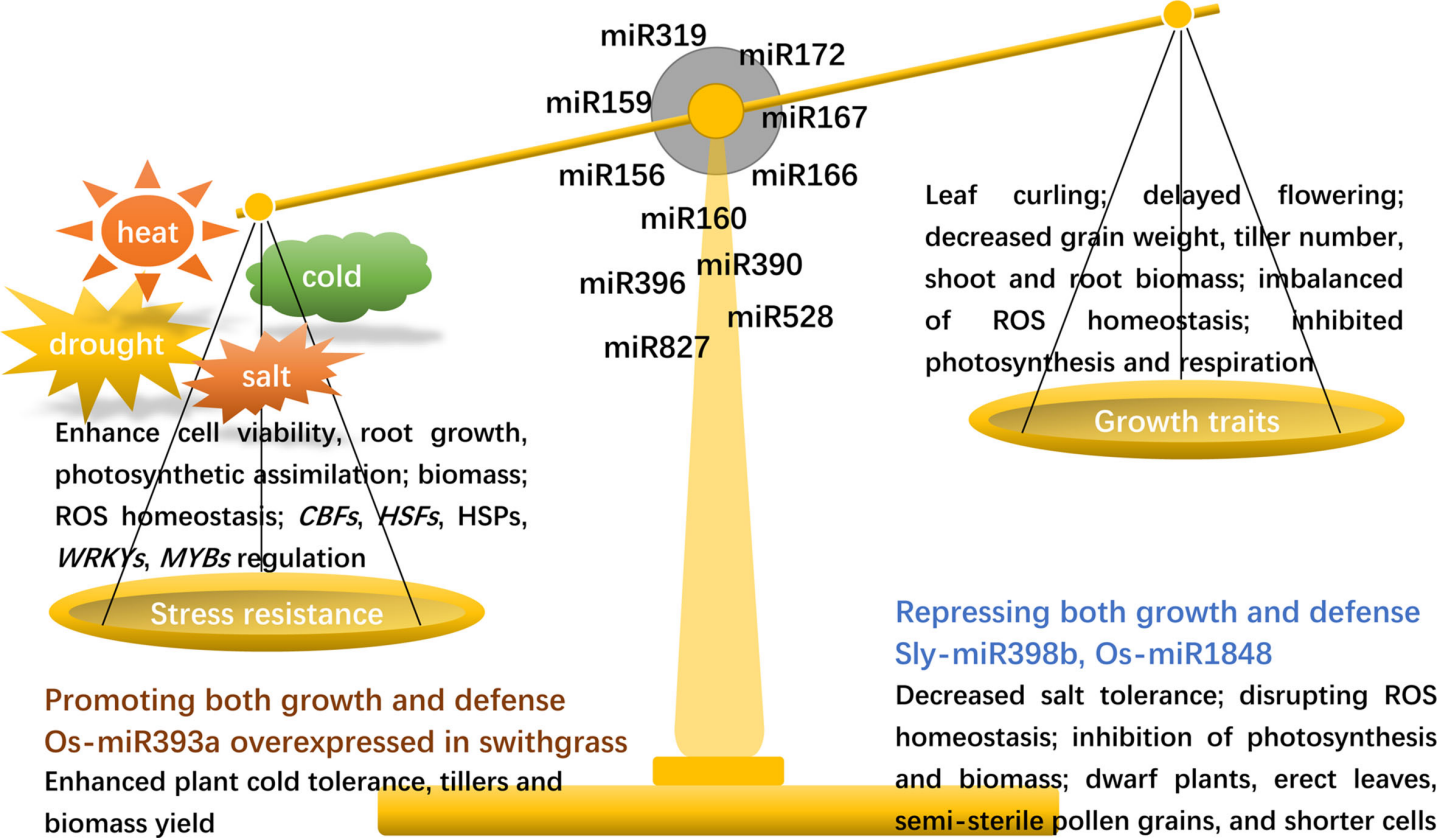
A diagram of miRNAs in regulating the trade-off between plant growth and development versus abiotic stress resistance. While improving plant abiotic resistance, miRNAs usually inhibit plant growth and development, affect plant morphological structure, suppress photosynthesis and respiration, disrupt ROS balance, and finally reduce crop yield. The interesting cases are that Os-miR393a promotes both growth and stress resistance while Sly-miR398b and Os-miR1848 repress growth and stress resistance. ROS, reactive oxygen species; *CBFs*, *C-REPEAT BINDING FACTORS*; *HSFs*, *HEAT STRESS TRANSCRIPTION FACTORS*; HSPs, heat shock proteins; *MYBs, MYOBLASTOSIS*.

The miR390-TAS3-ARF pathway is evolutionary conserved in land plants (Xia et al., 2017). In poplar (*Populus* spp.), the miR390-TAS3 module modulates lateral root growth under salt stress (He et al., 2018). Transgenic creeping bentgrass overexpressing Os-miR393a show improved drought resistance with reduced stomata density and a denser cuticle, enhanced thermotolerance with the activation of heat-shock protein genes, and improved salt tolerance with increased total chlorophyll contents and uptake of potassium, which may be at the cost of fewer tillers and lower biomass (Zhao et al., 2019). Interestingly, the heterologous expression of Os-miR393a in switchgrass (*Panicum virgatum* L.) enhances plant cold tolerance, tillers and biomass yield in both greenhouse and field tests (Liu et al., 2017). However, Os-miR393 overexpression leads to more tillers, early flowering and less tolerance to salt and drought in rice (Xia et al., 2012). The different roles of Os-miR393 in rice and creeping bentgrass may be explained by the divergent roles of the targets of Os-miR393 indifferent species. Moreover, transgenic creeping bentgrass overexpressing Os-miR396c or Os-miR528, which regulates the metabolic balance of ROS in plants by participating in diverse redox reactions, show enhanced salt tolerance accompanied with increased chlorophyll content, water retention and cell membrane integrity, enhanced capacity for maintaining K^+^ and ROS homeostasis, but display shortened internodes, reduced leaf area, leaf veins, and plant biomass (Yuan et al., 2015; Yuan et al., 2019). A recent publication shows that the disruption of the miR396-OsGRF4 module results in enlarged grains and enhanced cold tolerance in rice (Chen et al., 2019), suggesting that the precise editing of miRNAs targets may be an important approach to improve crop stress resistance without compromising growth and yield. Specifically, sly-miR398b is found to repress both growth and stress tolerance in tomato. Overexpression of sly-miR398b in tomato leads to inhibition of photosynthesis, reduced shoot and root biomass, and decreased salt tolerance along with the disruption of ROS homeostasis (He et al., 2021). Another miRNA, miR535, which belong to the miR156/529/535 superfamily, negatively regulates cold stress responses as well as rice seedling growth (Sun et al., 2020). Under well-watered and drought conditions, transgenic barley overexpressing ath-miR827, which was driven by the CAMV35S promoter, showed reduced water use efficiency, inhibited growth, delayed flowering, and decreased grain weight. In contrast, transgenic barley expressing Hv-miR827, driven by the drought-inducible promoter Zm-Rab17, showed no growth defects but possessed enhanced drought resistance with improved water use efficiency and a higher recovery rate after severe drought stress (Ferdous et al., 2017). These results imply that stress-inducible promoters such as Zm-Rab17, and the miRNAs Zm-miR156c and Hv-miR827 can be engineered to produce stress-tolerant plants without the sacrifice of growth or development. The miRNAs involved in this trade-off are presented in **Figure 1**.

Photosynthesis is one of the most severely affected processes during abiotic stress (Muhammad et al., 2021; Song et al., 2021b). For instance, the net photosynthesis rate, the actual quantum yield of photosynthesis and Fv/Fm are reduced, O_2_^• -^ is accumulated in sly-miR398b overexpression plants under salt stress, indicating that overexpression of sly-miR398b aggravates the photosynthesis reduction and triggers photoinhibition under salt stress (He et al., 2021).

Examination of the publications mentioned above illustrates the roles of many miRNAs and their targets in the trade-off between growth and abiotic stress responses. These miRNAs provide a basis for bringing about optimal agricultural production under various environmental stresses through molecular design breeding.

## siRNAs involved in the trade-off between growth and stress responses

Compared with our accumulated understanding of miRNAs involved in the trade-off between plant growth and abiotic stress responses, the specific roles of siRNAs in the trade-off, such as hc-siRNAs, phasiRNAs and nat-siRNAs, remain largely unknown.

Hc-siRNAs mainly participate in RdDM, which is required for maintenance of CHH methylation. The roles of hc-siRNAs in the trade-off between plant growth and development and abiotic stress responses remain largely elusive. In cucumbers (*Cucumis sativus* L.), cold stress induces a substantial and global impact on transposable element (TE)-related hc-siRNA-directed RdDM, which results in the demethylation of mCHH. Cold-induced differentially-methylated regions (DMRs) may be correlated with the transcription changes in ethylene biosynthesis-related CsACO3 and an *Arabidopsis* RAP2.4-like ethylene-responsive (AP2/ERF) transcription factor, which may contribute to the temperature-dependent sex determination in cucumber (Lai et al., 2017). In rose (*Rosa hybrida*), cold stress up-regulates mCHH levels in the promoter of *RhAG*, an AGAMOUS homolog, which may result in the repressed expression of *RhAG* and the cold-induced increase in petal number (Ma et al., 2015b). Cold stress-induced changes in the DNA methylation level in the promoter of *ALLANTOINASE* (*ALN*), a negative regulator of dormancy, is associated with seed dormancy in *Arabidopsis* (Iwasaki et al., 2019). In cotton anthers, heat stress disrupts genome DNA methylation, especially CHH methylation, in a heat-sensitive line but not in a heat-tolerant line, which may be related to microspore abortion and anther indehiscence (Min et al., 2014; Ma et al., 2018). However, the comprehensive function of hc-siRNAs in the above processes remains to be elucidated.

Among phasiRNAs, the role of tasiRNAs in the trade-off between development and abiotic stress responses has been reported. High temperature inhibits tasiRNAs biogenesis by reducing SGS3 protein content, which in turn relieves PTGS and contributes to heat-induced early flowering and attenuated immunity in *Arabidopsis* (Zhong et al., 2013; Liu et al., 2019a). In the miR390-TAS-ARF pathway, miR390 targets *TAS3* to produce tasiRNAs, which inhibit the expression of *ARF* genes, thereby regulating plant lateral root growth as well as drought stress tolerance (Marin et al., 2010; He et al., 2018; Wen et al., 2020). Although many miRNA-*PHAS* modules have been identified to be responsive to abiotic stress (Shuai et al., 2016; Sosa-Valencia et al., 2017; Yu et al., 2019; Wang et al., 2020), the roles of *PHAS*-derived phasiRNAs in the trade-off between plant growth, development and abiotic stress tolerance remain to be investigated.

Although there are many predicted NAT pairs in plants, only a small percentage of them can produce nat-siRNAs under normal conditions or in response to environmental or developmental stimuli. Several examples of NAT-derived nat-siRNAs have been reported in *Arabidopsis*. The NAT pair of *DELTA(1)-PYRROLINE-5-CARBOXYLATE DEHYDROGENASE* (*P5CDH*)-SRO5 generates nat-siRNAs that are involved in salt resistance (Borsani et al., 2005). As well, the base-pairing of *KOKOPELLI* (*KPL*) and *ARIADNE14* (*ARI14*) generate a sperm-specific NAT that produces *cis*-nat-siRNAs to regulate double fertilization (Ron et al., 2010). Interestingly, natural antisense transcripts of *MIR398b* and *MIR398c* suppress the biogenesis of miR398, thereby attenuating plant thermotolerance (Li et al., 2020d). As mentioned above, nat-siRNAs have been reported to modulate double fertilization, heat and salt stress responses (Borsani et al., 2005; Ron et al., 2010; Li et al., 2020d). Whether nat-siRNAs contribute to the growth-defense trade-off remains to be uncovered.

## LncRNAs participate in the trade-off between growth and stress responses

lncRNAs are a kind of non-coding RNAs with a length of more than 200 nt, which do not encode protein, but can act as decoy, signal, guide, or scaffold molecule to regulate the expression of protein-coding genes at the transcriptional and post-transcriptional levels (Wang and Chang, 2011). According to their position to adjacent genes, lncRNAs can be divided into antisense lncRNA, enhancer lncRNA, intergenic lncRNA, bidirectional lncRNA, and intronic lncRNA (Ponting et al., 2009). lncRNAs have a variety of biological functions, such as assisting alternative splicing, regulating chromosome structure, regulating translation, promoting or inhibiting mRNA degradation, and adsorbing miRNAs to regulate the functions of miRNA target genes (Ponting et al., 2009; Wang and Chang, 2011; Zhang et al., 2019b; Jha et al., 2020). Plant lncRNAs have been found to participate in the regulation of plant growth and development, and in the response to abiotic and biotic stresses (Chekanova, 2015; Yu et al., 2019; Jha et al., 2020; Tiwari and Lata, 2021).

Several plant lncRNAs have been reported to modulate the trade-off between growth, development, and abiotic stress responses. The most well-known lncRNAs are *COOLAIR* and *COLDAIR*, which originate from the genomic region of *FLOWER LOCUS C* (*FLC*) upon cold treatment and modulate the vernalization process required for flowering in some species. *COOLAIR* is induced by cold signal and target the 3’ end of *FLC* mRNA to degrade it during vernalization. *COLDAIR* is located in the first intron of *FLC* and can directly interact with CURLY LEAF (CLF) in the POLYCOMB REPRESSIVE COMPLEX 2 (PRC2) protein complex, recruiting the PRC2 complex to the *FLC* gene locus. PRC2, which catalyzes the trimethylation of lysine 27 of histone H3 (H3K27me3), inhibits *FLC* expression by enriching H3K27me3 and thus promotes flowering in *Arabidopsis* (Swiezewski et al., 2009; Heo and Sung, 2011; Kim and Sung, 2017; Kim et al., 2017). A lncRNA, *SVALKA*, is also induced by cold stress and represses the expression of *CBF1* in *Arabidopsis*, which provides a new mechanism to maximize cold tolerance with mitigated fitness costs (Kindgren et al., 2018). In potato, the central clock output transcription factor CYCLING DOF FACTOR 1 (StCDF1) and its lncRNA counterpart StFLORE modulate stomatal growth and diurnal opening to regulate water loss. Elevated expression of *StFLORE* transcripts increases drought tolerance, but delays tuber formation and reduces tuber number, suggesting the important roles of the *StCDF1-StFLORE* locus in the trade-off between vegetative reproduction and drought tolerance (Ramirez Gonzales et al., 2021).

The lncRNA DROUGHT-INDUCED LNCRNA (DRIR) in *Arabidopsis* is induced by drought, salt, and ABA and positively regulates salt and drought responses. Compared with wild-type, the growth and stomatal closure of *DRIR1*-overexpressing seedlings are more sensitive to ABA treatment, suggesting that DRIR1 may activate the ABA signaling pathway (Qin et al., 2017). Through systematic analysis of lncRNAs, circular RNAs (circRNAs), and miRNAs in response to heat stress in cucumbers, some lncRNAs are speculated to interact with miR9748 and regulate plant thermotolerance through IAA and ethylene signaling (He et al., 2020). In upland cotton, salt stress represses the expression of *lncRNA354*, which acts as a competing endogenous RNA of miR160b to modulate *GhARF17/18* genes. Silencing *lncRNA354* or *GhARF17/18* increased plant height and salt tolerance in cotton, while heterologous overexpression of *lncRNA354* or *GhARF17/18* in *Arabidopsis* decreased plant height and salt tolerance, suggesting that lncRNA354 may regulate plant growth and stress response by activating IAA signaling (Zhang et al., 2021). Overall, lncRNAs-mediated regulation of the trade-off between plant abiotic stress response and growth and development remains unclear. More efforts are needed to elucidate the interaction between lncRNAs, sRNAs, and mRNAs, and their regulatory roles in plant trade-off.

## Phytohormones involved in the ncRNAs−mediated trade-off between growth and stress responses

**Figure 2:**
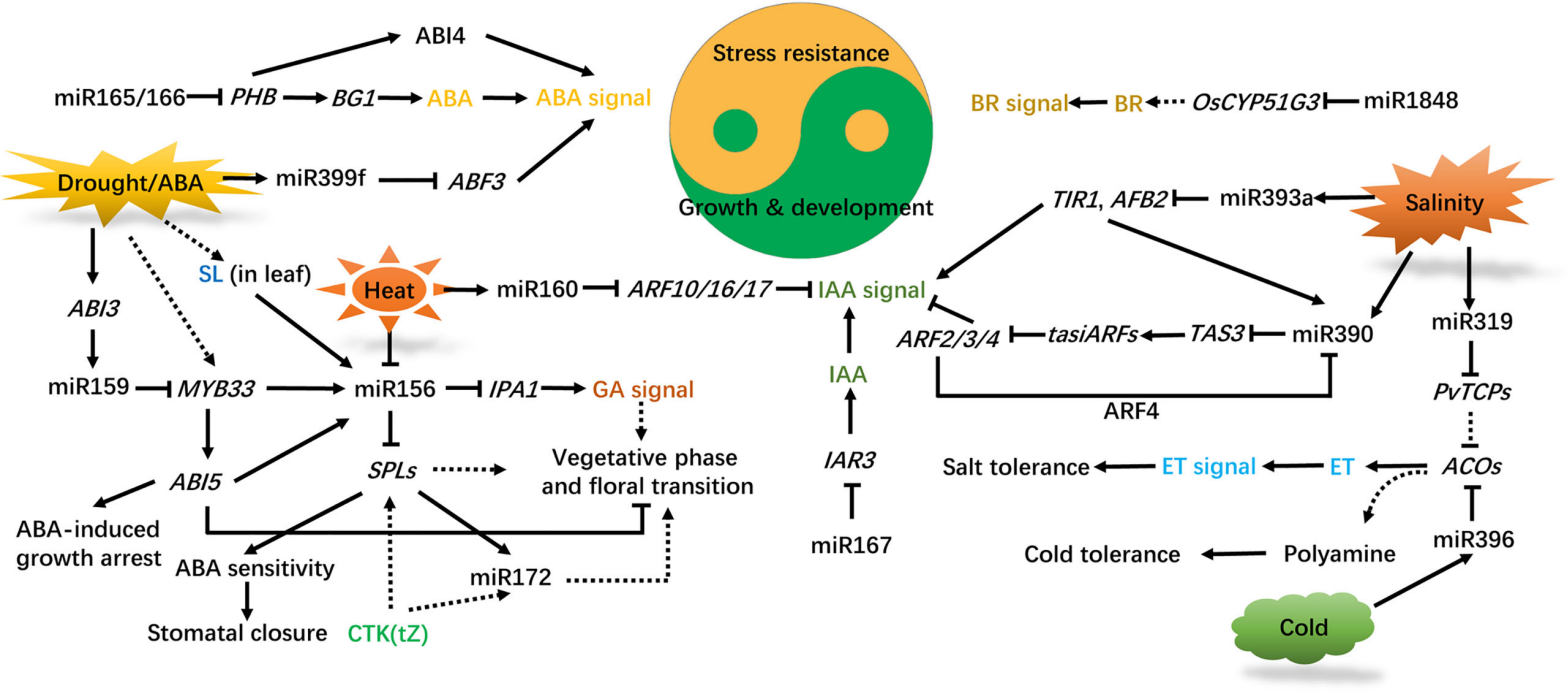
Interconnection of phytohormones and miRNAs in regulating plant development and abiotic stress responses. ABA synthesis is repressed by miR165/166, IAA accumulation is affected by miR167, BR synthesis is regulated by miR1848, and the ET content is influenced by miR319/396. Drought, heat, cold, and salinity affect plant growth, development, and stress adaption by regulating hormone signal transduction mediated by miRNAs. ABA, IAA, GA, CTK, ET, BR, SL are implicated in these processes. Solid and dashed lines indicate direct and indirect regulation, respectively, and arrows and blunted lines indicate facilitation and inhibition, respectively. ABA, abscisic acid; IAA, indole acetic acid; GA, gibberellic acid; CTK, cytokinin; ET, ethylene; BR, brassinosteroid; SL, strigolactone; *PHB*, *PHBULOSA*; *BG1*, *β-GLUCOSIDASE 1*; *ABI3/4/5*, *ABSCISIC ACID INSENSITIVE 3/4/5*; *ABF3*, *ABA-RESPONSIVE ELEMENT-BINDING TRANSCRIPTION FACTOR3*; *MYB33*, *MYOBLASTOSIS33*; *SPLs*, *SQUAMOSA PROMOTER BINDING-LIKEs*; *IPA1*, *IDEAL PLANT ARCHITECTURE1*; *TIR1*, *TRANSPORT INHIBITOR RESISTANT1*; *AFB2*, *AUXIN SIGNALING F-BOX*; *TAS3*, *TRANS-ACTING-SIRNA3*; *ARF4*, *AUXIN RESPONSE TRANSCRIPTION FACTOR4*; *IAR3*, *IAA-ALA RESISTANT3; PvTCPs*, *TEOSINTE BRANCHED1/CYCLOIDEA/PCFs* in *Panicum virgatum* L.; *ACOs*, *1-AMINOCYCLOPROPANE-1-CARBOXYLIC ACID OXIDASES*.

Phytohormones are endogenous signal molecules, which play important roles in plant development, metabolism and stress responses. By regulating the expression of target genes, ncRNAs affect the metabolism, distribution and perception of hormones, as well as participating in the regulation of plant hormone signaling pathways in development and stress responses (Li et al., 2020c; Singh et al., 2021). The phytohormones auxin, gibberellic acid (GA), abscisic acid (ABA), ethylene (ET), strigolactone (SL), cytokinin (CTK), brassinosteroid (BR), salicylic acid (SA) and jasmonic acid (JA) are involved in the regulatory pathway mediated by ncRNAs (Li et al., 2020c; Visentin et al., 2020; Yu and Wang, 2020; Barrera-Rojas et al., 2021; Singh et al., 2021; Werner et al., 2021). The expression of ncRNAs can be directly altered by plant hormones. For example, IAA enhances the expression of miR390 (Yoon et al., 2010). ABA induces the expression of miR159, miR393, and miR399 (Sunkar and Zhu, 2004; Reyes and Chua, 2007; Baek et al., 2016), but suppresses the expression of miR167 and miR319 (Liu et al., 2009). Here we summarize the roles of phytohormones, especially ABA and auxin, in ncRNAs (mainly miRNAs) −mediated trade-off between plant development and abiotic stress responses (**Figure 2**).

### Aba

ABA is a vital phytohormone that regulates bud dormancy, leaf abscission, stomatal closure, seed development, seedling growth and other physiological functions. As well, ABA is an important signal molecule for plants to quickly and accurately respond to abiotic stress such as drought, salt, and cold stress. Drought and salt stress can cause the accumulation of ABA, which in turn mediates the environmental adaptation of plants (Gong et al., 2020). Some miRNAs have been confirmed to function in the ABA signaling pathway. ABA and drought stress induce the expression of miR159, which reduces the sensitivity of germinated seeds to ABA by degrading the transcripts of the target genes *MYB101* and *MYB33*, thereby affecting seed germination and stress response through the ABSCISIC ACID INSENSITIVE 3 (ABI3)-dependent pathway (Reyes and Chua, 2007). MYB33 acts upstream of miR156 to affect the juvenile-adult transition of plants through the central ABA signaling regulator ABI5 through dependent and independent pathways (Guo et al., 2021). A reduction of miR165/166 levels releases the inhibited expression of its target gene *PHBULOSA* (*PHB*), which promotes the expression of BG1 (hydrolysis of abscisic acid-glucose ester to ABA) and ABI4, thus leading to the accumulation of ABA and activation of ABA signal transduction, drought and cold resistance (Yan et al., 2016). The transgenic *Arabidopsis* seedlings overexpressing gma-miR172c are sensitive to ABA, the result of which is an increased expression of *ABI3* and *ABI5*, reduced leaf water loss rate, an improved survival rate, and earlier flowering after drought and salt stress (Li et al., 2016). The loss-of-function double mutant of miR160 and miR165/166 displayed compromised leaf development and drought tolerance. The compromised leaf development may be explained by miR160-directed regulation of ARFs through auxin signaling, whereas the miR165/166-Class III homeodomain leucine zipper proteins (HD-ZIP IIIs) module may contribute to drought tolerance through ABA signaling (Yang et al., 2019). Overexpression of miR394a/b or mutation of its target gene *LEAF CURLING RESPONSIVENESS* (*LCR*) increases salt sensitivity and drought tolerance in an ABA-dependent manner in *Arabidopsis* (Song et al., 2013). The ABA-induced miR399f may regulate plant stress responses by degrading the target genes *ABA-RESPONSIVE ELEMENT-BINDING TRANSCRIPTION FACTOR3* (*ABF3*) and *CSP41b*, a chloroplast RNA binding protein (Baek et al., 2016). These reports suggest that miR156, miR159, miR172, miR165/166, miR394, and miR399 participate in plant development and stress tolerance through interaction with the ABA signaling pathway. As mentioned above, lncRNA DRIR may activate ABA signaling pathway to positively modulate salt and drought responses in *Arabidopsis* (Qin et al., 2017).

### Auxin

Auxin is another widely studied phytohormone involved in miRNA-mediated regulation of plant growth, development and environmental adaptation. The regulatory function of auxin mainly depends on the auxin signaling pathway which is composed of the auxin receptor F-box proteins TIR1/AFBs, AUX/IAA (auxin/indole-3-acetic acid) proteins, and ARFs (Vanneste and Friml, 2009). miR393 affects plant sensitivity to auxin signaling by negatively regulating the expression of the target genes *TIR1/AFBs* and modulates salt stress adaptation of *Arabidopsis* through regulating auxin signaling, the redox system and osmotic pressure (Iglesias et al., 2014; Chen et al., 2015). miR160, miR167 and miR390, which all target the ARFs, play important roles in plant growth and development. *Arabidopsis* miR160 affects the auxin signaling pathway by cleaving *ARF10* mRNA, and also interacts with the ABA signaling pathway, thereby affecting seed germination and postembryonic development (Liu et al., 2007). miR160 also inhibits anther dehiscence in cotton by inhibiting the expression of *ARF10/17* and activating the auxin signaling pathway under high temperature stress (Ding et al., 2017). miR390 targets *TAS3* to produce tasiRNAs that inhibit the expression of *ARF2/3/4*, which in turn repress miR390 through ARF4 (Marin et al., 2010). In poplar trees, miR390-TAS3-ARF4 maintains lateral root growth under salt stress through attenuating the inhibition of auxin signaling caused by salt toxicity (He et al., 2018). In rice, Os-miR393a is promoted while Os-miR390 is inhibited by stresses such as drought and salt, and Os-miR390-directed lateral root growth is negatively regulated by Os-miR393 (Lu et al., 2018), implying a mutual regulation between miRNAs in plant development and environmental adaptation. In upland cotton, lncRNA354 may modulate the trade-off between plant growth and abiotic stress response by activating IAA signaling (Zhang et al., 2021).

### Other plant hormones

BR can regulate plant stress response through interactions with ET, GA, IAA, CTK, ABA, JA and SA pathways (Kour et al., 2021). BR negatively regulates the miRNA-mediated translational repression of target genes by interfering with the distribution and localization pattern of the miRNA-responsive protein AGO1 on the endoplasmic reticulum (Wang et al., 2021). Os-miR1848 is found to repress rice growth and salt stress resistance through BR signaling. Transgenic rice plants overexpressing Os-miR1848, or carrying RNAi of the target gene *OsCYP51G3*, an obtusifoliol 14α-demethylase, is more sensitive to salt stress and displays typical phenotypic changes relating to phytosterol and BR deficiency, including shorter cells, erect leaves, dwarfism and semi-sterile pollen grains (Xia et al., 2015).

Os-miR319 positively regulates ET accumulation and salt tolerance in switchgrass. This salt tolerance is dose-dependent on ET content as well as through ET synthesis mediated by Os-miR319. The repression of *PvPCF5*, a target of Os-miR319 in switchgrass, results in enhanced ethylene accumulation and salt tolerance, which extends our understanding of the synergistic effects of the miR319-PvPCF5 module and ethylene synthesis in plant salt tolerance (Liu et al., 2019b). In trifoliate orange [*Poncirus trifoliata* (L.) Raf.], ptr-miR396b is induced by cold stress and guides the cleavage of 1-*AMINOCYCLOPROPANE-1-CARBOXYLIC ACID OXIDASE* (*ACO*) mRNA. ptr-miR396b positively regulates cold tolerance through repressing ethylene synthesis while simultaneously promoting polyamine synthesis, which ultimately results in enhanced ROS scavenging (Zhang et al., 2016).

SL has been proved to act as a molecular link between miR156 and drought responses in tomato. SL is sufficient for the induction of miR156 under both normal conditions and drought stress. miR156 inhibits the expression of *SPL* and promotes stomatal closure in response to ABA, suggesting the important roles of SL-miR156 module in stomatal behavior during the recovery stage after drought stress (Visentin et al., 2020).

SA is reported to induce the expression of *AtR8* lncRNA in germinating seeds, and the loss of *AtR8* lncRNA inhibits seed germination under salt stress. NaCl treatment inhibits the expression of *AtR8* lncRNA in germinated seeds and further the germination of *atr8*, which has reduced accumulation of *AtR8* lncRNA. This report indicates that *AtR8* lncRNA plays an important role in salt stress during seed germination of *Arabidopsis* and SA may be implicated in this regulation (Zhang et al., 2020).

CTK regulates the proliferation and differentiation of plant cells, and widely participates in physiological and metabolic activities inside plant cell, and plant stress resistance (Mandal et al., 2022). The expression of miR172 is positively regulated by CTK in *Arabidopsis* (Werner et al., 2021), and gma-miR172 enhances plant drought and salt tolerance (Li et al., 2016). Therefore, CTK may improve plant salt and drought resistance through the action of miR172.

Above all, ncRNAs affects auxin, BR, ABA, ET, SL, SA and CTK signaling, and the biosynthesis and action of many ncRNAs are modulated by hormones, thereby illustrating the interconnectedness of plant growth and abiotic responsive hormonal regulatory machinery. ncRNAs may modulate plant growth and abiotic stress responses through regulating any step of phytohormone signaling, including the biosynthesis and transport of hormones, the activation of receptor/co-receptor complexes, the transmitting and amplifying hormone signals, and downstream targets of hormone signaling pathways. The detail roles of phytohormones in ncRNAs-mediated trade-off between growth and abiotic stress responses remain to be investigated.

## ncRNAs-mediated transgenerational stress memory in the trade-off between growth and stress responses

Upon challenge by abiotic stress, some stress-induced physiological, metabolic, or molecular changes in stressed plants can be mitotically maintained for the duration of plant’s life. This phenomenon is known as somatic stress memory. Some changes are meiotically transmitted to the next generation only, whereas some stress memory can be maintained through at least two subsequent non-stressed generational offspring. These effects are referred to as intergenerational and transgenerational stress memory, respectively (Heard and Martienssen, 2014; Kinoshita and Seki, 2014; Lamke and Baurle, 2017; Liu and He, 2020; Sadhukhan et al., 2022). Transgenerational memory may offer the non-stressed progeny plants the ability to respond quickly and strongly to recurrent stress. Recent studies have revealed that some epigenetic modifications, including DNA methylation, and histone methylation and acetylation play important roles in the regulation of plant transgenerational stress memory (Lang-Mladek et al., 2010; Ou et al., 2012; Zheng et al., 2017; Preite et al., 2018; Cong et al., 2019; Liu et al., 2019a). Currently, the functional studies of ncRNAs in plant stress transgenerational memory are very limited.

In *Arabidopsis*, heat stress activates the retrotransposition of a copia-type retrotransposon named *ONSEN*. This effect can also be observed in the non-stressed plants with compromised siRNA biogenesis, suggesting a crucial role for the siRNA pathway in preventing transgenerational stress memory (Ito et al., 2011). Surprisingly, genes nearby the *ONSEN* insertions acquire a responsiveness to heat stress, which may generate novel heat-responsive regulatory machineries (Ito et al., 2011). The progeny of UV-C stressed plants displays decreased leaf number, late flowering, and increased transposon expression, which depends on the activity of DCL2-4 (Migicovsky and Kovalchuk, 2014). Whether DCL proteins regulate transgenerational stress memory through modulating the biogenesis of siRNAs remains unclear. Bra-miR168 and its target *braAGO1* were found to participate in the stress-induced transgenerational memory in *Brassica rapa* (Bilichak et al., 2015). Although these reports suggest the possible roles of small RNAs in transgenerational stress memory, the detailed functions of small RNAs in the transgenerational memory of trade-off between growth and abiotic stress responses remain largely unknown. Interestingly, prolonged heat stress can induce the transgenerational memory of early flowering and attenuated immunity in *Arabidopsis* (Liu et al., 2019a). Heat-induced activation of HEAT SHOCK TRANSCRIPTION FACTOR A2 (HSFA2) and the H3K27me3 demethylase RELATIVE OF EARLY FLOWERING 6 (REF6) form a feedback loop to suppress the biogenesis of tasiRNAs, which releases the expression of tasiRNA target *HEAT-INDUCED TAS1 TARGET 5* (*HTT5*) to drive early flowering and compromised immunity. Thus, heat stress induces transgenerational inheritance of phenotypical changes through a cooperative epigenetic network involving transcription factors, histone demethylation and tasiRNAs, thereby ensuring successful reproduction and stress adaptation (Liu et al., 2019a). The roles of small RNAs in the establishment, maintenance, or erasing of plant transgenerational stress memory of trade-off clearly need to be further investigated.

## Concluding remarks and future perspectives

In the past two decades, ncRNAs have been shown to play important roles in plant development and environmental adaptation. Some ncRNA-based technologies have been developed to protect plants from environmental stresses. First, clustered regularly interspaced short palindromic repeat/CRISPR-associated proteins (CRISPR/Cas) are used for targeted miRNA modification, including knock-out of *MIR* genes, up- or down-regulation of target genes by CRISPR activation, or CRISPR interference systems (Deng et al., 2022). Secondly, spray-induced gene silencing (SIGS) is an environmentally friendly technique which uses synthesized dsRNA to target pathogenic factors to inhibit diseases (Wang and Jin, 2017). SIGS can also be used in plant abiotic stress adaptation. SIGS of *VvGST40*, a glutathione S-transferase gene, improves resilience after drought stress in grapevine (*Vitis vinifera* L.) (Nerva et al., 2022). Thirdly, artificial microRNAs (amiRNAs) are sRNA molecules that target one or more specific genes, and can efficiently and specifically inhibit the expression of the target. For example, the proline dehydrogenase coding gene *StProDH1*, a target of miR6461, regulates proline accumulation under drought stress in potato. Artificial microRNA-mediated gene silencing of *StProDH1* enhances potato drought tolerance (Li et al., 2020b). Using amiRNAs technology with OsMIR528 as backbone to knockdown *OsBEAR1* results in reduced salt stress tolerance in rice (Teng et al., 2022). As well, a short tandem target mimic (STTM) can be used to inhibit the function of miRNAs in plants in responses to abiotic stresses (Zhang et al., 2018b; Li et al., 2020a). Moreover, virus-induced gene silencing (VIGS) has been widely used for functional analyses in plant abiotic stress tolerance such as drought and salt (Ramegowda et al., 2014). RNA interference can also be used to inhibit gene expression. miPEPs, which are encoded by short ORFs in pri-miRNAs, mainly enhance the activity of their associated miRNAs by increasing their accumulation and hence downregulating the target genes. miPEPs are proved to regulate plant growth, development, and metabolism (Yadav et al., 2021), and can be considered as novel and effective tools to investigate the interaction between plants and environment. All of the above techniques can be used to study the function of ncRNAs in regulating plant growth and responses to environmental stresses and improve stresses tolerance and agronomic traits in crops.

With the progress of sequencing technology and bioinformatic analysis tools, more and more ncRNAs have been discovered. In-depth exploration of the roles of ncRNAs in the trade-off between growth and development, and environmental adaptation can provide theoretical and technical guidance for crop improvement. As listed in **Box 1**, there are still many questions which deserve consideration in the future. Important progress has been achieved on the interaction between sRNAs and lncRNAs in plant disease resistance (Zhou et al., 2020). However, the regulatory networks underlying ncRNAs in plant responses to abiotic stress remain rather limited. The natural environmental stress usually induces multiple changes, such as ion toxicity and nutrient imbalance caused by salt stress. 21 miRNAs in soybean roots could respond to salt stress or phosphorus deficiency separately, or the combination of this two stresses (Ning et al., 2019). Further investigation on the roles of ncRNAs in plant responses to multiple stresses should be paid more attention. Interestingly, *Pseudomonas putida* affects the growth, development, and stress response of *Arabidopsis thaliana* by affecting the biogenesis and action of miRNAs in the root system (Jatan et al., 2020). The roles of ncRNAs in plant interaction with commensal microbes under adverse environmental conditions need to be investigated in future studies.

The improvement of plant stress resistance is often accompanied by plant growth defects and deterioration of yield traits. Therefore, uncoupling plant abiotic stress resistance and growth inhibition is the primary issue that needs to be addressed before the application of ncRNAs through transgenic technologies. Stress-inducible promoters can be utilized to eliminate growth inhibition caused by constitutive expression of defense genes. Furthermore, the results from the laboratory cannot fully reflect the effects of ncRNAs in the complex environment in a field. Thus, transgenic lines with excellent stress tolerance and growth traits obtained from the laboratory need to undergo years of multi-point tests to ensure the persistence and penetrance of the beneficial effects. Further studies should also be centered on the ncRNAs-mediated transgenerational stress memory to provide new insight for using ncRNAs to develop crops with improved yield and abiotic stress resistance.

**Box 1 Future research directions**

How ncRNAs coordinate plant responses to multiple abiotic stresses?
How to uncouple plant stress resistance from growth inhibition?
How to efficiently identify the key ncRNAs responsible for a plant’s acclimation to abiotic stresses?
What is the role of ncRNAs in the establishment, maintenance and erasing of plant transgenerational stress memory?
How to quickly identify a suitable stress-inducible promoter to induce the expression of ncRNAs or their targets to promote growth and abiotic stress tolerance?
How are plant hormones fine-tuned by abiotic stresses?
How to improve plant resistance to abiotic stress through transgenerational stress memory?
How to quickly determine functional lncRNAs to accelerate investigation on the roles of lncRNAs in abiotic stress responses in crops?

## Author contributions

The original draft was prepared by YiZ, YeZ, and FC, and edited by WZ, JL and FC. All authors contributed to the article and approved the submitted version.

## Funding

This work was supported by funding from the Natural Science Foundation of Shanghai (22ZR1455100 to YiZ), the National Natural Science Foundation of China (32070564 to JL), and Natural Science Foundation of Yunnan Province (202101AW070002 and 202201AT070090 to JL, 2019FB031 to FC).

## Acknowledgments

Due to space limitations, we apologize to our colleagues whose important work are not cited in this review.

## Conflict of interest

The authors declare that the research was conducted in the absence of any commercial or financial relationships that could be construed as a potential conflict of interest.

## Publisher’s note

All claims expressed in this article are solely those of the authors and do not necessarily represent those of their affiliated organizations, or those of the publisher, the editors and the reviewers. Any product that may be evaluated in this article, or claim that may be made by its manufacturer, is not guaranteed or endorsed by the publisher.

## Abbreviations

SPLs, Squamosa Promoter Binding-likes; IPA1, ideal plant architecture1; WD40, Encoding WD40 repeat; MYBs, Myoblastosis; TCPs, Teosinte branched1/cycloidea/pcfs; ARF10/16/17, Auxin response transcription Factors; HD-ZIP III, Homeodomain leucine zipper III; IAR3, IAA-ALA Resistant3; NF-YA, Nuclear transcription Factor y subunit alpha; AP2, Apetala2; IDS1, Indeterminate Spikelet1; TOE1/2, Target of eat1/2; TAS3, Trans-acting-sirna3; AFB/TIR1, Transport inhibitor resistant1/auxin signaling f-box; GRF, Growth-regulating factor; bHLH, Basic helix-loop-helix; ACO, 1-Aminocyclopropane-1-carboxylic acid oxidase; CSD1/2, CU/ZN-Superoxide dismutase; CCS1, Copper chaperone of CSD1; AGL51/52/78, Agamous-like 51/52/78; PHO2, Phosphate 2; LAC3/LAC12/LAC13, Laccase 3/12/13; AO, Amine oxidase; PPO, Polyphenol oxidase; AAO, Ascorbate oxidase; POD, Peroxidase; SOD, Superoxide dismutase.
