# Supplementary material for: Non-coding RNAs fine-tune the balance between plant growth and abiotic stress tolerance
Source: Front Plant Sci. 2022 Oct 12;13:965745. doi: 10.3389/fpls.2022.965745 (PMC9597485; doi:10.3389/fpls.2022.965745)
Supplement: Supplementary file 1 [file Presentation_1.pptx]

## Slide 1
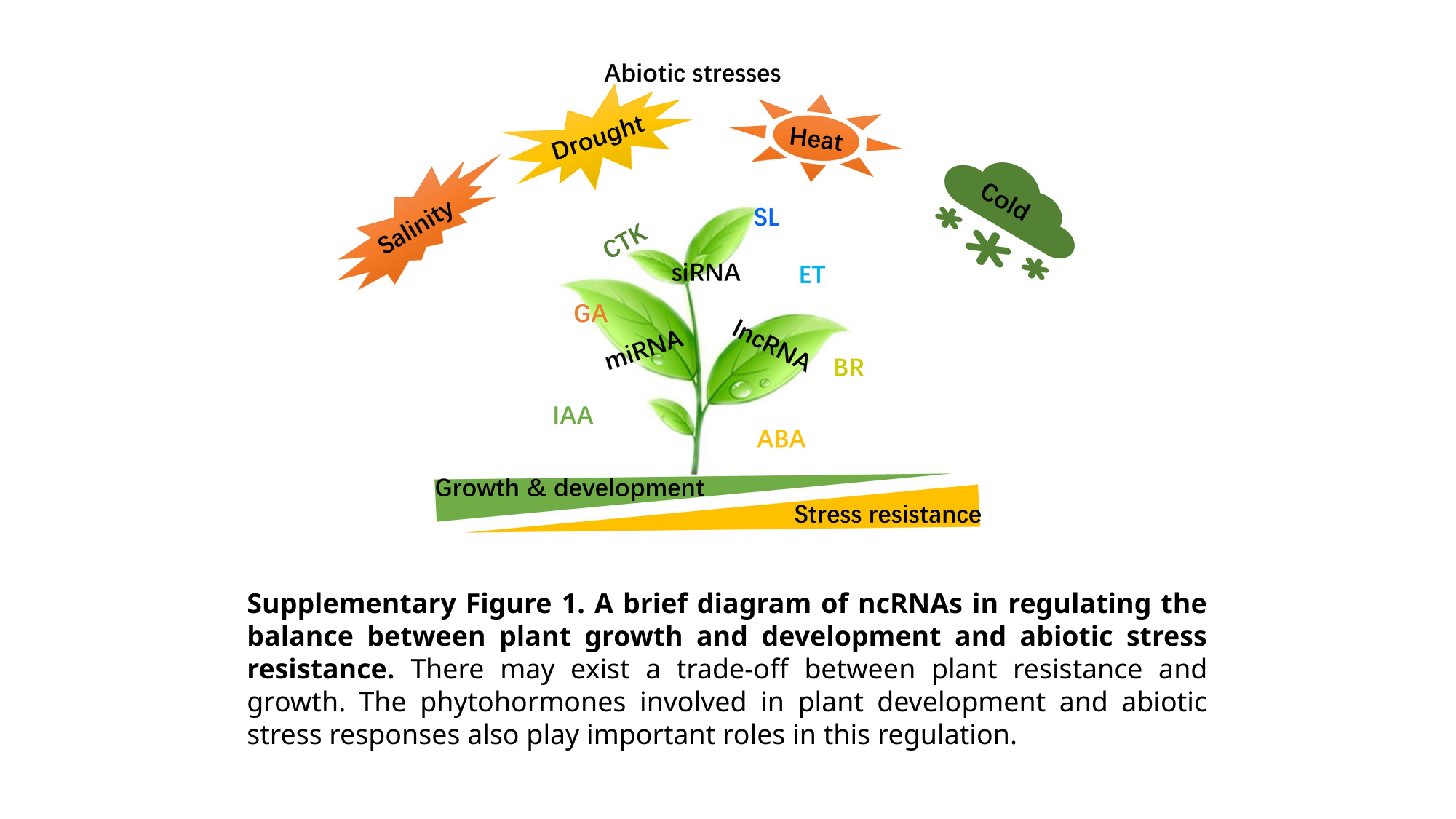

Supplementary Figure 1. A brief diagram of ncRNAs in regulating the balance between plant growth and development and abiotic stress resistance. There may exist a trade-off between plant resistance and growth. The phytohormones involved in plant development and abiotic stress responses also play important roles in this regulation.
